# Bird Diversity, Birdwatching Tourism and Conservation in Peru: A Geographic Analysis

**DOI:** 10.1371/journal.pone.0026786

**Published:** 2011-11-23

**Authors:** Liisa Puhakka, Matti Salo, Ilari E. Sääksjärvi

**Affiliations:** Department of Biology, University of Turku, Turku, Finland; Australian Wildlife Conservancy, Australia

## Abstract

In the face of the continuing global biodiversity loss, it is important not only to assess the need for conservation, through e.g. gap analyses, but also to seek practical solutions for protecting biodiversity. Environmentally and socially sustainable tourism can be one such solution. We present a method to spatially link data on conservation needs and tourism-based economic opportunities, using bird-related tourism in Peru as an example. Our analysis highlighted areas in Peru where potential for such projects could be particularly high. Several areas within the central and northern Andean regions, as well as within the lowland Amazonian regions of Madre de Dios and Loreto emerge as promising for this type of activity. Mechanisms to implement conservation in these areas include e.g. conservation and ecotourism concessions, private conservation areas, and conservation easements. Some of these mechanisms also offer opportunities for local communities seeking to secure their traditional land ownership and use rights. (Spanish language abstract, Abstract S1).

## Introduction

The target set in 2002 by the Conference of the Parties to the Convention on Biological Diversity to achieve a significant reduction in the rate of biodiversity loss by the year 2010 has not been met [1]. Although protected area networks have grown substantially during the last decades they are still incomplete worldwide [2] and failed to halt the biodiversity crisis [3]. However, establishing new protected areas through traditional government-led procedures is not the only feasible means of conservation in many places where biodiversity is threatened, and sometimes it is not applicable at all. Thus, in addition to gap analyses identifying areas or species in need of further conservation, it is important also to look for other, perhaps more practical solutions for protecting biodiversity through creating local conservation initiatives.

One such approach is to promote conservation through environmentally responsible and socially sensitive tourism. Nature and biodiversity themselves function as tourism attractions both for a general tourist market and for more specified niche markets such as whale- and birdwatching tours. Globally, most of the high-priority areas for biodiversity conservation are also key regions for tourism development, and tourism has been growing particularly in biodiversity hotspots in the South [4]. While not a universal cure-for-all, sustainable forms of tourism can provide alternative livelihoods and incentives for communities to protect valuable habitats in key localities, given the right circumstances and proper planning [5].

Spatial studies integrating data on conservation effort, distribution of key taxa, and tourism activity are required to identify such areas. We used Peru and a specialized end of nature-based tourism, birdwatching tourism, as an example to demonstrate a method to integrate these different types of data. Peru makes for an excellent subject for this type of investigation: the species richness, especially that of birds, is among the highest in the world [6], [7], while the country also is an established tourism destination. There is definitely need for further conservation work in Peru: while the Neotropics and particularly Amazonian lowlands enjoy the highest protected area coverage on Earth [2], the Amazonian reserves should by no means be assumed to at present capture all Amazonian species [8]. In addition, the Tropical Andes has been identified as one of the world's most significant gap species areas, i.e. areas with the greatest occurrences of species not represented in any protected area [9].

The main aim of the present article was to present a method to pinpoint areas and sites with high potential for integrated tourism and conservation, using birdwatching tourism in Peru as an example, and to discuss available mechanisms for promoting local conservation initiative through nature-based tourism. We have approached this aim by analysing various data sets concerning conservation as well as the distribution of important bird species and that of bird-related tourism in Peru. Even though our focus in this article was birdwatching tourism, it needs to be stressed that the role of this specific niche of tourism as an aid for conservation has to be seen as part of a wider whole of nature-based and cultural tourism. We see the strength of bird-based tourism as providing an extra argument for conservation, not it being a panacea for conservation and poverty alleviation.

## Materials and Methods

**Figure 1:**
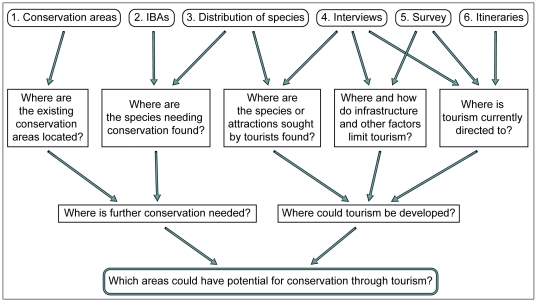
Data used in the study and their relation to the main and auxiliary study questions.

We used the following data sets: 1) map of the conservation area network [10], [11], [12], 2) map of the Important Bird Area (IBA) network [13], [14], 3) distribution maps of Peruvian bird species (all species, as well as only those of conservation concern and endemics) [7], [15], 4) interviews of key informants, 5) a survey among birdwatchers, and 6) itineraries of tour companies specialized in birdwatching. The data cover the scale from applied conservation science (data sets 1 and 2) through pure species distribution data (data set 3) to applied tourism study (data sets 4 to 6). The ways in which these different data relate to the specific study questions are visualised in Figure 1. We explain the structure of the data and the methods of analysis in the following sections separately for each data source. We used ArcGIS software (version 10) to carry out all spatial analyses.

### Data sets

#### Conservation and IBA networks

The Peruvian national system of protected areas (*Sistema Nacional de Áreas Naturales Protegidas por el Estado*, SINANPE) in its current form was first established in 1990 and as of June 2011 it consisted of a total of 72 nationally administered protected areas. In addition to the areas included in the SINANPE, in June 2011 there were 13 regional and 34 private conservation areas. The protected area network is maintained by the National Service of Natural Protected areas (*Servicio Nacional de Áreas Naturales Protegidas por el Estado*, SERNANP), designated in 2008 under the Ministry of the Environment. Altogether these 119 areas cover ca. 21.2 million ha, approximately 16.5% of the total area of Peru [10]. We obtained a shapefile for the protected area network of Peru from the World Database on Protected Areas [12] and updated it to match the current situation [11].

BirdLife International uses the concept of Important Bird Areas (IBAs) to define key sites for conservation. In the Americas, IBAs are selected based on the presence of 1) bird species of global conservation concern, 2) assemblages of restricted-range bird species, 3) assemblages of biome-restricted bird species, or 4) globally important congregations of birds [16]. In Peru, the process for identifying IBAs was initiated in 2003, and the first final list consisting of 128 IBAs was published in 2005 [13]. An updated list was published in 2009. The current 116 IBAs cover a total area of ca. 20 million ha (16% of the Peruvian territory) [14].

We obtained a shapefile for the current IBA network by digitizing the maps by Franke et al. [13] and Angulo Pratolongo [14] – for areas where the IBA network was not changed in the new revision, we used maps digitized from the more detailed maps in Franke et al. [13], and for the areas where protected areas were designated in their entirety as IBAs, we copied them from the above-mentioned shapefile for protected areas. To be able to assess the coverage of the current protected area network of Peru, we separated the IBA areas not included in the conservation area network using an overlay analysis.

#### Distribution maps of Peruvian bird species

We studied the distributions of Peruvian bird species to assess both the need for conservation in different areas and the distribution of resources for birding tourism – the birds themselves – throughout the country. We focused on three aspects: number of bird species of conservation concern, number of endemic bird species, and total bird species richness. We obtained from Chicago Field Museum the polygon shapefiles used to create the distribution maps for the Birds of Peru field guide [7], [15], and extracted from there distribution data for all Peruvian bird species classified in the IUCN categories critically endangered (CR: 7 spp.), endangered (EN: 31 spp.) and vulnerable (VU: 62 spp.), and for all endemic species (101 spp. [7]). We included only the land area of Peru, and omitted all purely pelagic species. The total number of species included in the analysis was thus 1664, approximately 91% of the bird species of Peru [17]. The number of species of conservation concern included in our analysis was 75 and that of endemic species 99, with 31 species being both endemic and of conservation concern.

In order to visualize the overlap of the distributions of the species, we converted the polygon shapefiles containing the distribution of each species to raster files (cell size 0.05 decimal degrees, or ca. 5.5 km), where the cell values were 1 in the cells that belonged to the distribution area of the species, and 0 elsewhere. We then summed the cell values of the resulting rasters separately for the species of conservation concern, the endemic species, and all species, creating three raster files whose cell values (theoretically 0–75 for species of conservation concern, 0–99 for endemic species, and 0–1664 for all species, in practice 0–13, 0–29 and 0–666, respectively) depict the number of bird species' ranges overlapping in the area of each cell (RASTER1, RASTER2 and RASTER3).

#### Interviews of key informants

Key informant interviews provide a general view on the situation of birdwatching tourism in Peru. We interviewed representatives from tourism companies, experts in Peruvian ornithology, and bird guides working in Peru. These interviews were carried out in Lima, Cusco and Iquitos in 2006 as part of the Master of Science thesis of the first author [18]. The interviews were semi-structured and were conducted in Spanish and in English, according to the preference of each interviewee. The length of the interviews varied between 20 and 60 minutes, the average length being 40 minutes. We recorded the interviews using an MD recorder (Sony Walkman MZ N707) and later transcribed them.

In the first part of the interview, we asked the interviewees to identify areas they thought were important birdwatching tourism destinations in Peru at the moment, and those they believed would be important destinations in the future. For this, we provided each interviewee with a map of Peru in the scale 1:4,000,000, in which region boundaries, major roads, rivers and cities were depicted as symbols. The map was created with assistance of the *Centro de Datos para la Conservación* at the National Agrarian University La Molina in Lima (CDC-UNALM). No place names were printed on the map, but a reference map in the scale of 1:2,500,000 was available for the interviewees. There were seven of these main interviews where the interviewee was asked to draw areas on a map, one being focused solely on the Loreto Region.

We digitalized by hand the areas drawn by the interviewees, comparing the drawings with the interviewees' verbal descriptions of the areas to monitor for any errors in their placement. If the interviewee had mentioned a name of a locality but had drawn the corresponding area to a wrong place on the map, we moved the corresponding polygon so that the locality mentioned by the interviewee was in the centre, maintaining the original shape and size. We copied protected areas mentioned by the interviewees in their entirety from the above-mentioned protected area data. We depicted specific localities such as cities, towns or rainforest lodges as point data, and sought coordinates for them using a gazetteer provided by the National Geospatial-Intelligence Agency (NGA, http://earth-info.nga.mil/gns/html/namefiles.htm), Google Earth and web searches. Similarly, we depicted stretches of roads or rivers as line data, copying them from the original map data used to create the base map for the interviews, or if not depicted there, digitizing them by hand using as a model a map of Peru in the scale of 1:2,500,000. We then created buffers of 5 km for the point and line data, and added these to the respective polygon data.

We created a summary raster map of the areas identified by the interviewees similarly as was described previously for the bird species distributions. However, since only six of the seven interviewees covered the whole area of Peru and one only Loreto, we converted the cell values separately for Loreto and the rest of Peru to percentages reflecting the portion of interviewees including each area to their drawing (RASTER4).

The interviews included also a second part with open-ended questions about the key informants' views on the current situation and future of birdwatching tourism in Peru. Two additional interviews consisted only of these open-ended questions. The total number of key informants interviewed was therefore nine.

#### Survey among birdwatchers

In order to analyse the demand for birdwatching tourism and what birdwatchers themselves look for in a destination, we conducted a survey among birdwatchers in September 2009. We created the survey using a free online survey service (www.esurveyspro.com), and announced it in the beginning of September on three e-mail lists specializing in birdwatching in Peru and in the Neotropics – Birdingperu, INCASPIZA and NEOBIRD. Birdingperu is a mailing list for people interested in birdwatching in Peru, maintained by the owner of a Peruvian birdwatching tour company. INCASPIZA is aimed primarily towards conservation of the birds of Peru, and is maintained by a Peruvian association under the same name. NEOBIRD is for bird observations in the Neotropics, and is maintained by the University of Houston. We made the survey available in both English and Spanish, and accepted replies for one month. We sent one reminder of the survey to the three e-mail lists roughly one week before time for participation in the survey ended.

In the questionnaire, we asked the respondents to list the most important bird species of Peru in three categories (top five species per category): 1) the respondent's most precious bird sightings in Peru: five bird species the respondent has seen in Peru that rank highest in their personal life list, 2) Peruvian bird species the respondent would most want to see, and 3) Peruvian bird species the respondent believes could be most important for promoting tourism in the country. We asked the respondents also to name three strengths and three weaknesses Peru in their view has as a birdwatching destination compared to other countries. The survey included also questions on the respondents' experience in birding in Peru, other Latin American countries and elsewhere, as well as some background questions, and an opportunity to include additional comments.

We received a total of 47 usable entries: 37 through the English and 10 through the Spanish version of the survey. The vast majority of the respondents were male (41, females 6), and the most common age was between 31 and 40 years (13) (Table 1). US citizens were the largest nationality group (18), followed by Peruvians (16) (Table 2). Of those respondents who were not permanent residents of Peru, 12 had spent less than one month in the country, 11 over six months, six one to six months, and one respondent had never been to Peru. All but two of the respondents had participated in a bird-related activity in Peru, the most common of these being casual or independent birdwatching (32) (Table 3).

**Table 1:** Age of the respondents to the survey for birdwatchers.

Age group	Respondents
21–30 years	5 (10.6%)
31–40 years	13 (27.7%)
41–50 years	10 (21.3%)
51–60 years	10 (21.3%)
61–70 years	6 (12.8%)
71 years or above	3 (6.4%)

Numbers and percentages of respondents belonging to each age group.

**Table 2:** Nationality and country of residence of the respondents to the survey for birdwatchers.

Country	Nationality	Country of residence
USA	18 (38.3%)	19 (40.4%)
Peru	16 (34.0%)	18 (38.3%)
Costa Rica	0	1 (2.1%)
Ecuador	0	1 (2.1%)
South Africa	1 (2.1%)	1 (2.1%)
Belgium	1 (2.1%)	1 (2.1%)
Denmark	1 (2.1%)	1 (2.1%)
Finland	1 (2.1%)	1 (2.1%)
Germany	1 (2.1%)	0
Great Britain	2 (4.3%)	1 (2.1%)
Netherlands	2 (4.3%)	1 (2.1%)
Norway	2 (4.3%)	1 (2.1%)
Spain	1 (2.1%)	2 (4.3%)
Sweden	1 (2.1%)	0

Numbers and percentages of respondents of each nationality and country of residence.

**Table 3:** Experience level of the respondents to the survey for birdwatchers.

Activity	In Peru	Elsewhere in Lat.America	Elsewhere
Has birdwatched casually or independently	32 (68.1%)	30 (63.8%)	29 (61.7%)
Has been a customer on a birdwatching tour	24 (51.1%)	18 (38.3%)	18 (38.3%)
Has participated in scientific bird studies	22 (46.8%)	9 (19.1%)	18 (38.3%)
Has been a guide on birdwatching trips	17 (36.2%)	8 (17.0%)	12 (25.5%)
Has been a customer on a general tour featuring birds	7 (14.9%)	8 (17.0%)	8 (17.0%)

Numbers and percentages of the respondents that had taken part in the mentioned activities.

We analysed the distributions of the bird species receiving most votes in the three categories (top 10 for each category). For these species, we extracted the distribution maps [7], [15] and created a raster map of the overlap of the ranges (RASTER5). In addition, we examined the most important sites to sight each of these species in Peru using a birdwatching guidebook [19]. We collected all localities the guide listed for each species, plotted them on a map and created a summary raster map using the same methods as described previously (RASTER6). We included both these maps as separate rasters, as even though they represent the same group of species, they represent different aspects of these species' ranges – the distribution maps represent the whole distribution of the species, while the localities mentioned in the birdwatching guidebook [19] are chosen taking into account their reachability, and thus highlight specific known locations where the species can with relative certainty be sighted.

We analysed the respondents' answers to the questions about the strengths and weaknesses of Peru as a birdwatching destination by identifying individual issues mentioned by the respondents and categorizing these under general themes. For each individual issue and general theme, we noted the number of respondents mentioning it in their answers.

#### Itineraries of tour companies specialized in birdwatching

We carried out an analysis of the tours offered by companies specializing in birdwatching tourism in Peru to create an overview of the market offer for birdwatching tourism and the most important birdwatching tourism destinations in Peru. The criteria for selecting companies to include in this analysis were the following: to qualify, a company had to 1) be focused on birds (The company expresses in its webpage and advertising that birds are the primary focus of the company's tours, and tours specializing in other attractions are at most a secondary focus.), 2) be focused mainly on tours within Peru, 3) arrange tours to the whole country (The company is not tied to for example a particular lodge.), 4) be based in Peru, 5) have in its web pages detailed itineraries of the tours offered by the company, and 6) be relatively easy to find by a potential client (The company's web pages are relatively easy to find by standard web search tools.). We focused the analysis on tour companies based in Peru, omitting any international companies, because the aim was to identify areas which tour operators specialized in Peru have found to be the most important destinations. It is unlikely that the sets of destinations offered by international tour companies are more extensive than those offered by in-country operators.

The criteria narrowed the analysis down to 5 companies. We read through the itineraries offered by these companies in their web pages, collected from them all sites and areas where the itineraries indicated that birds would be observed, plotted them on a map and created a summary raster map depicting the areas most visited by tour companies, using the same methods as described previously (RASTER7). Our aim was to analyse which sites were mentioned by each company, not in how many tours any given site was mentioned by each company. We viewed the “popularity” of a site thus only based on how many of the companies included it in their itineraries, not based on how many separate tours of each company included it.

### Creating a summary of the data sets

In order to identify areas where several of these different data suggest a high potential for conservation through tourism, we combined all seven raster datasets mentioned in the previous sections. We divided the cell values of each raster by their maximum cell value, thus rescaling them to contain values between 0 and 1. We then summed these rescaled rasters together. The resulting map shows where the maximums of different data sets coincide. Since the different data sets reflect different issues related to tourism and conservation opportunities, an area where several maximums coincide can be interpreted to have high opportunities for conservation through tourism. We gave all data sets the same weight: a location with the highest total number of species (666) is in our analysis equally valuable as a location visited by all tour companies.

Our analysis included only the land area of Peru, and thus omitted all purely pelagic bird species. Because of this, the potential of the coastal areas of Peru as sites for birdwatching tourism may be underrepresented in this study. We also assessed only the variation of total bird species richness, not the complementarity between different areas. Systematic conservation planning through e.g. irreplaceability analyses is arguably a useful tool to design an optimal conservation area network [20]. However, our aim was to pinpoint the areas with highest potential for this type of conservation activity, and a high concentration of species of interest is essential for this.

One critical aspect related to the nature of the data sets we used should also be noted. While for example accessibility to sites is a necessary consideration for any tourism project, we opted not to include in our analysis any data set representing pure accessibility or any other similar aspect not directly representing tourism potential or need for conservation. This type of data could only affect the results by excluding otherwise interesting areas, or by highlighting areas with no actual potential for tourism or need for conservation in the form of attractions or species of interest. However, accessibility is indirectly represented in at least three of the data sets we used: the areas mentioned by the interviewees, the sites mentioned in the birdwatching guidebook [19] and the itineraries of tour companies. We also compared the areas highlighted in our study with a data set representing accessibility [21], and address this comparison in the discussion.

## Results

### Where is further conservation needed?

**Figure 2:**
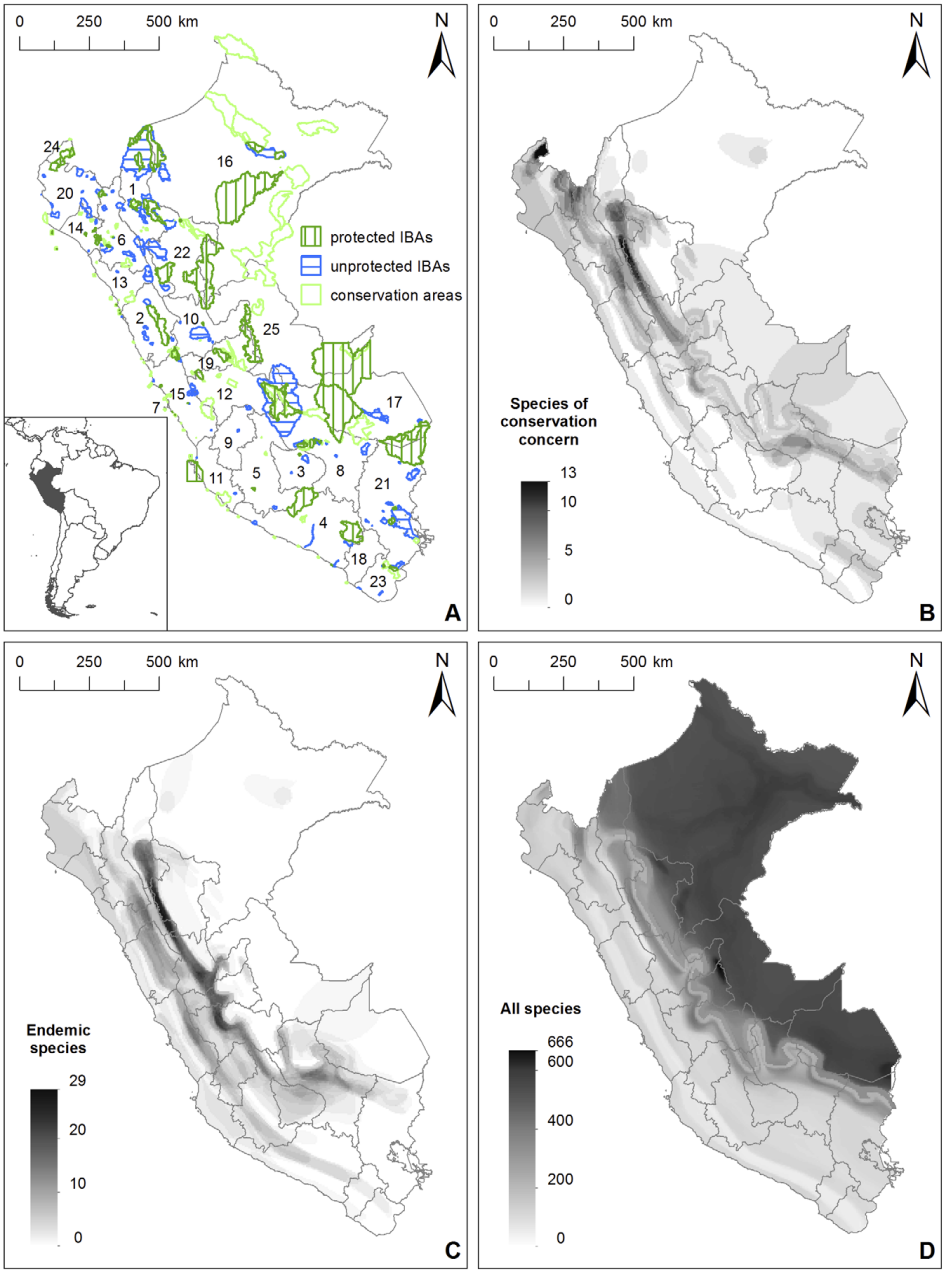
The Peruvian conservation area and IBA networks and distributions of Peruvian bird species. A) The location of Peru within South America (inset map, source: thematicmapping.org), the first-level administrative subdivision of Peru (25 regions: 1. Amazonas, 2. Ancash, 3. Apurímac, 4. Arequipa, 5. Ayacucho, 6. Cajamarca, 7. Callao, 8. Cusco, 9. Huancavelica, 10. Huánuco, 11. Ica, 12. Junín, 13. La Libertad, 14. Lambayeque, 15. Lima, 16. Loreto, 17. Madre de Dios, 18. Moquegua, 19. Pasco, 20. Piura, 21. Puno, 22. San Martín, 23. Tacna, 24. Tumbes, 25. Ucayali), and the overlap between the Peruvian conservation area and IBA networks. Overlap of the distributions of B) bird species belonging to the IUCN categories *critically endangered*, *endangered* and *vulnerable*, C) endemic bird species, and D) all Peruvian bird species.

The largest conservation areas in Peru are found in the lowland Amazonian regions of Madre de Dios, Ucayali and Loreto. Several of the largest conservation areas within these regions are designated as IBAs, such as the Alto Purús and Manu National Parks in Madre de Dios, Ucayali and Cusco, and the Pacaya Samiria National Reserve in Loreto. IBAs not included in the Peruvian conservation area networks are found especially in the northwestern parts of the country, from the region of Piura down to Huánuco (Figure 2, map A).

The areas with highest numbers of bird species of conservation concern are found in the northwestern parts of the country – the border areas between Peru and Ecuador in the regions of Cajamarca and Piura, with the highest numbers (13) found in eastern parts of the Tumbes region. Another concentration of bird species of conservation concern is found along the eastern slopes of the Andes from the region of Amazonas through San Martín, La Libertad, Huánuco, Pasco, Junín and Ayacucho to the Urubamba area in Cusco region, and further down to southeastern Peru, to the Peru-Bolivia border in the Puno region (Figure 2, map B). Endemic bird species were similarly most abundant along the eastern slopes of the Andes from Amazonas down to the Urubamba area in the Cusco region, and in slightly smaller numbers on the High Andes or on the western slopes from the region of Cajamarca through La Libertad and Ancash to eastern parts of the Lima region (Figure 2, map C). The Marañón area along the border between the regions of San Martín and La Libertad emerged as an area with high numbers of both bird species of conservation concern and endemic bird species.

A very different pattern is found in total bird species richness. The highest total numbers of bird species are found in the lowland Amazonian parts of Peru, with the highest peak (666 species) found in the eastern parts of Huánuco and Pasco, along their border with the Ucayali region. Additional peaks (600+ species) are found in the Peru-Bolivia border in the Madre de Dios and Puno regions, and in central Loreto, near the town of Iquitos (Figure 2, map D).

While the largest protected areas of Peru are located in the eastern, lowland Amazonian parts of Peru in Loreto, Ucayali and Madre de Dios, the highest abundance of both bird species of conservation concern and endemic bird species are found on the slopes of the Andes. A significant concentration of both endemic species and species of conservation concern but with apparently scarce protection is found on both sides of the Marañón Valley in the Regions of Cajamarca, Amazonas, San Martín and La Libertad. In central Peru, protected areas are scarce especially in the Huánuco Region. The northern border of Peru, especially the northern parts of Piura, Cajamarca and to some extent Amazonas also emerged as areas with high numbers of species of conservation concern but scarce protection. The San Martín – La Libertad border area with high numbers of both bird species of conservation concern and endemics corresponds roughly to two IBAs: Laguna de los Cóndores (PE062) and Río Abiseo y Tayabamba (PE066), of which the latter is partially covered by the Río Abiseo National Park.

### Where could tourism be developed?

#### Where are the species or attractions sought by tourists found?

**Figure 3:**
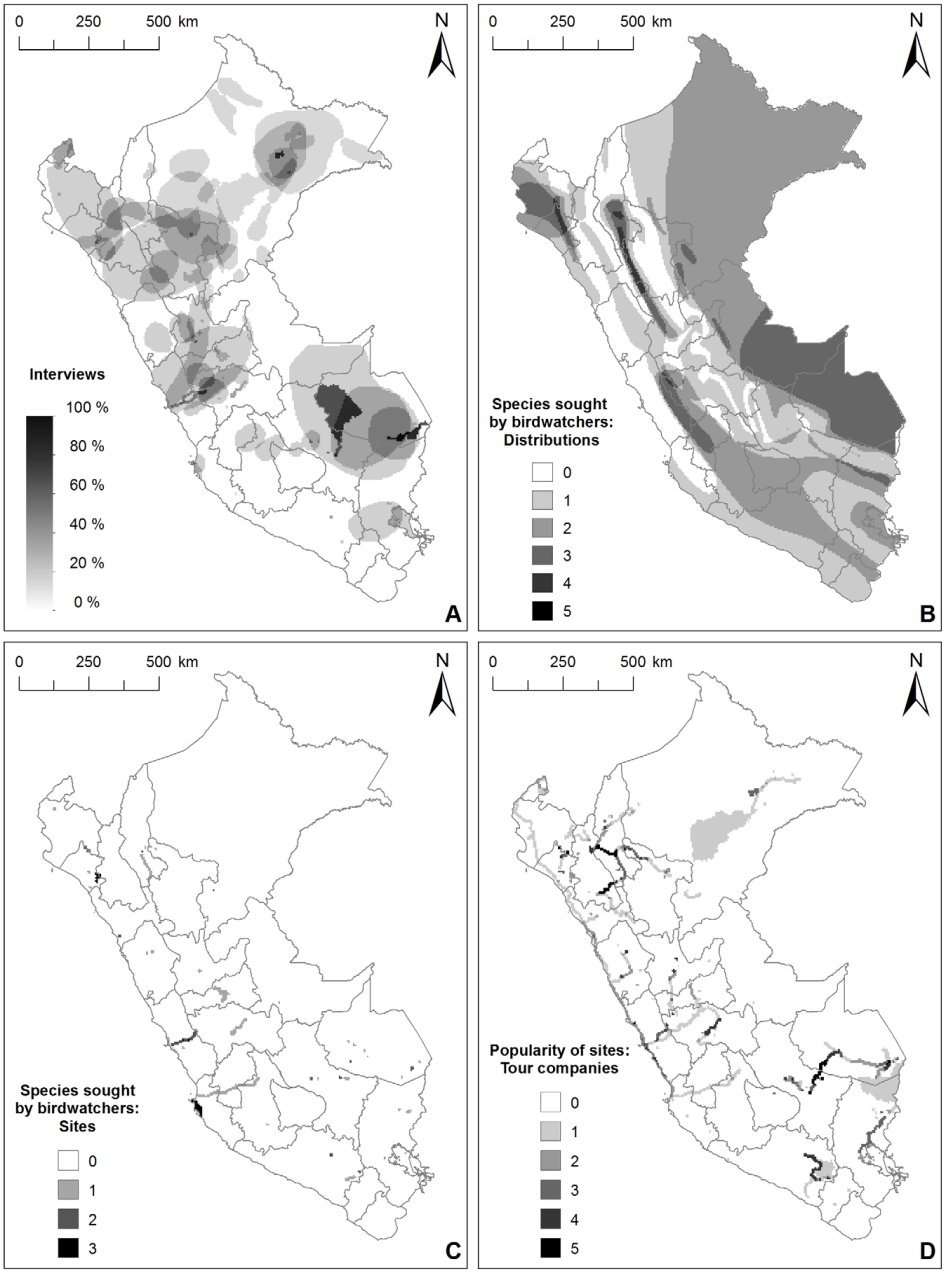
Distribution of birdwatching tourism opportunities and their current usage in Peru. Overlap of A) the areas drawn by the interviewees, B) the distributions of the species receiving most votes in the survey for birdwatchers, C) the locations where the species receiving most votes in the survey can be observed, and D) the destinations of tour companies organizing birdwatching tours in Peru.

The interviewees provided with a map were asked to draw areas which according to them are or could be important for birdwatching tourism in Peru. As we superimposed these drawings, five clusters of areas emerged: southeastern Peru (Cusco and Madre de Dios), central Peru, northern Peru, and the areas around Tumbes and Iquitos (Figure 3, map A). Of these, southeastern Peru does not host many endemic species, but is important from a birdwatching point of view due to its sheer species richness. Central Peru was also mentioned to have large numbers of species. Northern Peru was identified as an area with a high number of endemic bird species. For Loreto it was mentioned that easily accessible areas have suffered clearly visible human impact and in order to see more intact nature, one has to travel to areas far away from cities.

In the survey for birdwatchers, the respondents named a total of 206 different species in the three categories. The top ten for the three lists consisted of a total of 19 species (Table 4). These species include both spot endemic species such as the Long-whiskered Owlet (*Xenoglaux loweryi*) and Junín Grebe (*Podiceps taczanowskii*), and more widespread species such as the Harpy Eagle (*Harpia harpyja*). Several respondents also mentioned a charismatic, widespread species group in general, such as large parrots or hummingbirds. The highest overlap in the distributions of the 19 species is found within the Marañón area along the borders between Amazonas, San Martín and La Libertad (5 species: Andean Condor *Vultur gryphus*, Marvelous Spatuletail *Loddigesia mirabilis*, Pale-billed Antpitta *Grallaria carrikeri*, Andean cock-of-the-rock *Rupicola peruvianus* and Golden-backed Mountain-tanager *Buthraupis aureodorsalis*). Other areas with high overlap (4 species) are found in Lambayeque and Piura in the north, and in Pasco and Junín near the border of the Lima region in central Peru (Figure 3, map B). The locations mentioned in the birdwatching guidebook [19] with highest numbers of these species to be sighted (3) were Chaparrí in northwestern Peru, Paracas in Ica, and the so-called Central Highway in the Lima region (Figure 3, map C).

**Table 4:** Species receiving most votes in the survey for birdwatchers.

1. Personal favourites (126 spp.)	votes	2. Hopes to see (125 spp.)	votes	3. Promotional species (65 spp.)	votes
Andean Condor *Vultur gryphus* (NT)	14	**Marvellous Spatuletail ** ***Loddigesia mirabilis*** (EN)	17	**Marvellous Spatuletail ** ***Loddigesia mirabilis*** (EN)	29
**Marvellous Spatuletail ** ***Loddigesia mirabilis*** (EN)	14	**Long-whiskered Owlet ** ***Xenoglaux loweryi*** (EN)	15	Andean Cock-of-the-Rock *Rupicola peruvianus* (LC)	28
Harpy Eagle *Harpia harpyja* (NT)	10	**Scarlet-banded Barbet ** ***Capito wallacei*** (VU)	10	Andean Condor *Vultur gryphus* (NT)	22
Andean Cock-of-the-Rock *Rupicola peruvianus* (LC)	8	Harpy Eagle *Harpia harpyja* (NT)	9	**White-winged Guan** *Penelope albipennis* (CR)	13
**White-winged Guan** ***Penelope albipennis*** (CR)	7	Andean Cock-of-the-Rock *Rupicola peruvianus* (LC)	8	Humboldt Penguin *Spheniscus humboldti* (VU)	12
Diademed Plover *Phegornis mitchellii* (NT)	6	**Golden-backed Mountain-tanager** ***Buthraupis aureodorsalis*** (EN)	8	**Long-whiskered Owlet ** ***Xenoglaux loweryi*** (EN)	12
Inca Tern *Larosterna inca* (NT)	6	**Junín Grebe** *Podiceps taczanowski* (CR)	7	**Junín Grebe** *Podiceps taczanowski* (CR)	10
**White-bellied Cinclodes ** ***Cinclodes palliatus*** (EN)	5	Andean Condor *Vultur gryphus* (NT)	4	Scarlet Macaw *Ara macao* (LC)	9
Humboldt Penguin *Spheniscus humboldti* (VU)	4	**White-winged Guan ** ***Penelope albipennis*** (CR)	4	Inca Tern *Larosterna inca* (NT)	8
**Peruvian Plantcutter ** ***Phytotoma raimondii*** (EN)	4	Humboldt Penguin *Spheniscus humboldti* (VU)	3	Harpy Eagle *Harpia harpyja* (NT)	7
**Tumbes Tyrant ** ***Tumbezia salvini*** (NT)	4	**Pale-billed Antpitta ** ***Grallaria carrikeri*** (LC)	3		
		Peruvian Recurvebill *Simoxenops ucayalae* (NT)	3		
		Titicaca Grebe *Rollandia microptera* (EN)	3		

The top 10 species of each category and the species' conservation status by IUCN categories (LC: *least concern*, NT: *near threatened*, VU: *vulnerable*, EN: *endangered*, and CR: *critically endangered*). Species written in **bold** are endemic to Peru.

#### What limits tourism?

In the survey for birdwatchers, we asked the respondents to mention three strengths and three weaknesses they think Peru has as a birdwatching destination (Tables 5 and 6). Unsurprisingly, the rich nature was named as Peru's strength by nearly all of the respondents (43). Most of these (37) mentioned the birds themselves, with specifically the total number of bird species mentioned by 28 of the respondents, and endemic species by 17. Another high-ranking strength under this theme was habitat diversity (28). Good tourism infrastructure and services provided to the tourists were the second-largest general theme, with mentions from 23 respondents. Of these, 16 mentioned the ease of access to birding sites, although five of these with the caveats “in certain areas”, or “in the main tourism areas”. Cultural attractions and the possibility to combine birding with visits to archeological sites were mentioned by 14 respondents.

**Table 5:** Strengths of Peru as a birdwatching tourism destination.

Strengths	Respondents
1. Rich nature	43 (91.5%)
*Good birds in general*	*37 (78.7%)*
*Diversity of habitats*	*28 (59.6%)*
*Total number of bird species*	*28 (59.6%)*
*Endemic birds*	*17 (36.2%)*
*Undisturbed habitats*	*4 (8.5%)*
*Conservation areas*	*3 (6.4%)*
*Species unknown to science*	*2 (4.3%)*
*Spectacular landscapes*	*2 (4.3%)*
2. Tourism infrastructure and services	23 (48.9%)
*Easy access/good travel infrastructure*	*16 (34.0%)*
*Good facilities (lodges etc.)*	*4 (8.5%)*
*Information available (field guides etc.)*	*4 (8.5%)*
*Services: guides, tours, birding routes*	*4 (8.5%)*
3. Cultural attractions, combination of birding and culture	14 (29.8%)
4. Particular areas or attractions	7 (14.9%)
5. Safety	6 (12.8%)
6. Local knowledge	2 (4.3%)
7. Price	2 (4.3%)

Numbers and percentages of the respondents in the survey for birdwatchers mentioning each issue. Each general theme is numbered, and specific issues related to that theme (if any) are listed below it in *italics*. Note that the numbers of the specific issues do not necessarily add up to the number of respondents mentioning the general theme in question, since a respondent might have mentioned several individual issues within the theme or only the general theme itself.

**Table 6:** Weaknesses of Peru as a birdwatching tourism destination.

Weaknesses	Respondents
1. Tourism infrastructure and services	33 (70.2%)
*Poor road infrastructure and problems in access, transport issues*	*10 (21.3%)*
*Lack of good accommodation*	*8 (17.0%)*
*Lack of good guides*	*7 (14.9%)*
*Lack of available information*	*2 (4.3%)*
*Lack of promotion*	*2 (4.3%)*
*Independent travelling difficult*	*2 (4.3%)*
*Language requirements*	*2 (4.3%)*
2. Security issues	30 (63.8%)
*Crime, terrorism and corruption*	*17 (36.2%)*
*Health issues*	*5 (10.6%)*
*Unreliable tourism operators*	*2 (4.3%)*
3. Natural/geographical conditions	11 (23.4%)
*Distances within country*	*6 (12.8%)*
*Rough nature*	*3 (6.4%)*
*Distance of country*	*2 (4.3%)*
4. Conservation issues, environmental degradation and litter	9 (19.1%)
*Habitat loss*	*4 (8.5%)*
*Pollution, litter*	*3 (6.4%)*
5. Local participation	7 (14.9%)
*Lack of opportunities for local participation, lack of financing etc.*	*4 (8.5%)*
*Lack of local birders*	*3 (6.4%)*
6. Issues in government agencies, lack of participation from government	4 (8.5%)
7. Price	4 (8.5%)
8. Poverty	2 (4.3%)

Numbers and percentages of the respondents in the survey for birdwatchers mentioning each issue. Each general theme is numbered, and specific issues related to that theme (if any) are listed below it in *italics*. Note that the numbers for the specific issues do not necessarily add up to the number of respondents mentioning the general theme in question, since a respondent might have mentioned several individual issues within the theme or only the general theme itself.

The most commonly named weaknesses were those related to tourism infrastructure and services provided for the tourists with mentions from 33 respondents. Problems in access due to poor road infrastructure or transport were mentioned by 10 of these, lack of accommodation by eight, and lack of good guides by seven. Security was the second largest general theme, with mentions by 30 respondents. Crime, terrorism or corruption was mentioned by 17 of these. Health issues such as food poisoning or tropical diseases were mentioned by five and the unreliability of especially local tourism operators by two respondents. Natural or geographical conditions such as distances within the country and rough natural conditions restricting traveling were brought up by 11 respondents. Conservation issues were highlighted by nine respondents. Of these, four mentioned specifically habitat loss due to e.g. social issues such as poverty or the growing population, or to make way for agriculture and mining. Another issue that was brought up was the minimal participation among Peruvians – four of the respondents mentioned that there are few opportunities for local participation due to e.g. lack of funds, and three mentioned in general the lack of local birders, and that there is minimal organization among them.

According to the interviewees, areas of southeastern Peru are traditionally the most important and well-known tourism destinations. This is reflected in the area's strong infrastructure, and abilities to receive large numbers of tourists. The area has high possibilities for both cultural and natural tourism and their combinations. Central Peru has the advantage of being accessible from Lima via road, which cuts down travel costs. Infrastructure was mentioned to be weak, with accommodation possibilities restricted mainly to cities. On the other hand, the price level was mentioned to be low in this area. It was mentioned that the area is not visited by great numbers of tourists. The infrastructure in north Peruvian destinations was mentioned to be weak, though somewhat better than in central Peru. For both Loreto and Tumbes it was mentioned that issues in transportation may limit tourism, since the areas are somewhat isolated. Especially for the Iquitos area, the scarcity and unreliability of flights to the city was mentioned to be a significant problem, although the situation seems to have recently improved.

#### Where is birdwatching tourism currently directed to?

We investigated the destinations which are currently important for birdwatching tourism in Peru both through the interviews and through an analysis of the itineraries of five tour companies organizing birdwatching trips in Peru (Figure 3, map D). In the interviews, as mentioned in the previous chapters, five main areas were highlighted as having the most important destinations. The analysis of the itineraries highlighted partly the same areas, however with some differences.

In southeastern Peru, the destinations of the Regions of Cusco and Madre de Dios – the Machu Picchu ruins, the so-called Manu road and Puerto Maldonado coincided with the areas highlighted in the interviews. These destinations were in fact represented in the itineraries of all five companies. Even though Lake Titicaca was mentioned by at least half of the interviewees, also areas of Puno away from the lake itself emerged as highly used destinations by the tour companies, with the stretch from the lake to Sandia Valley represented in more than half of the companies' itineraries. Colca valley and its well-known condor-watching site, *Cruz del Cóndor*, were mentioned by more than half the interviewees, but in addition to this, the itinerary data highlighted also the route from Colca to the city of Arequipa and Lake Salinas area east of the city.

In central Peru, areas mentioned by the interviewees and highlighted in the itinerary data coincided to a large extent with those used most by the tour companies. Areas included in all five companies' itineraries were the coastal destinations north and south of the city of Lima: Lomas de Lachay, Villa Marshes and Pucusana. Although not depicted in the map, pelagic trips starting from Lima were also included in the itineraries of three of the companies.

In northern Peru, the areas between Bagua, Pomacochas and Tarapoto, the Marañón valley area and the stretch between Cajamarca and Celendín in the Regions of Cajamarca, Amazonas and San Martín, as well as areas near Olmos and Abra Porculla on the border of Lambayeque and Piura were included in all five tour companies' itineraries. The areas in the central Amazonas Region, near Nuevo Salem and Imacita were also included in more than half the companies' itineraries. These are mostly known and visited for the spot endemic Orange-throated Tanager (*Wetmorethraupis sterrhopteron*). In Tumbes, only the Tumbes Mangrove reserve was included in more than half the companies' itineraries, and in Loreto, only the Allpahuayo-Mishana reserve. Additional individual destinations included in more than half the tour companies' itineraries were the Lake Llanganuco and San Damián in the Ancash Region, and Chao in La Libertad.

### Which areas could have potential for conservation through tourism?

**Figure 4:**
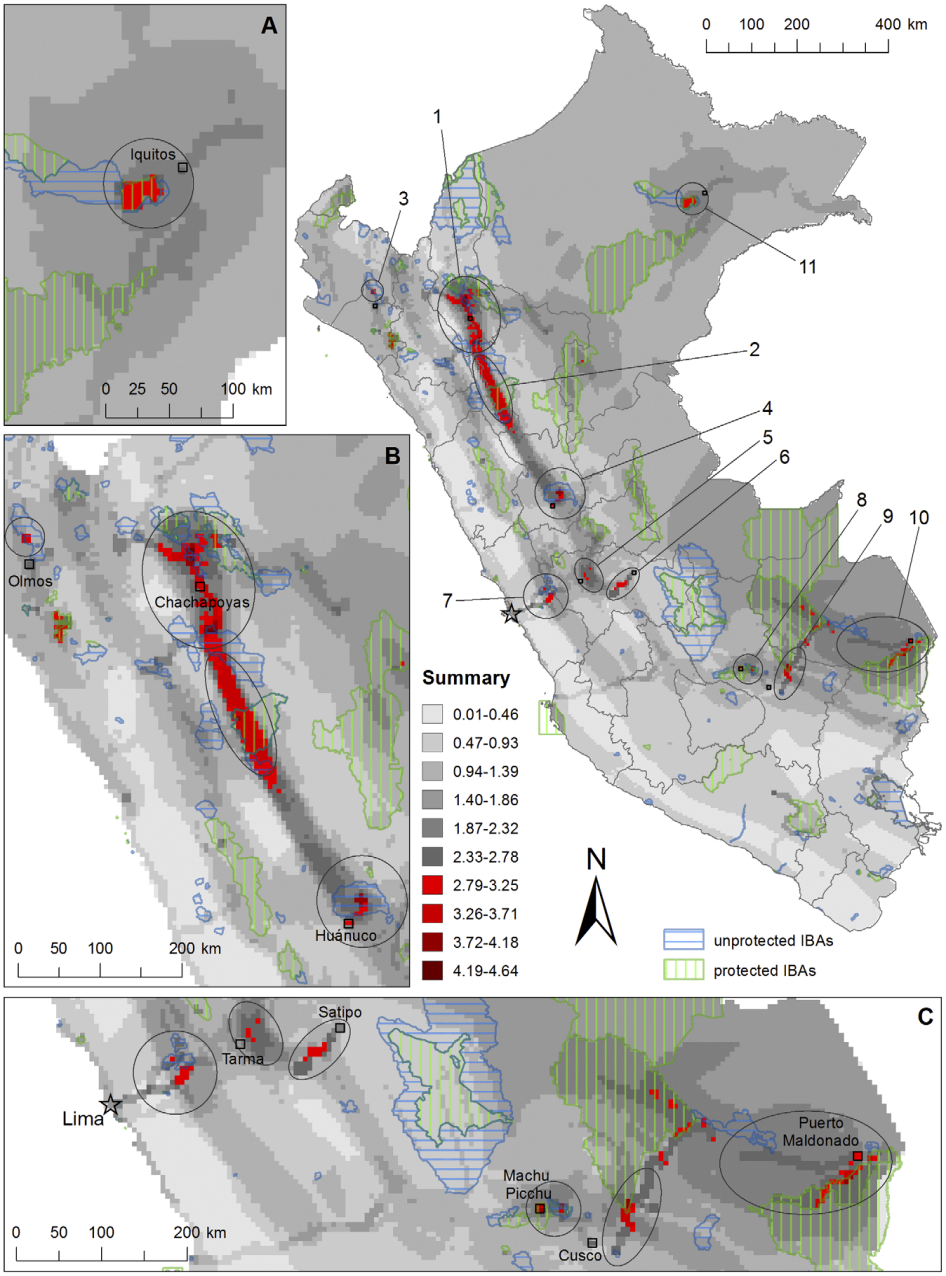
Areas of high potential for conservation through birdwatching tourism in Peru. Summary of Figure 2, maps B–D and Figure 3, maps A–D created by rescaling each map to values between 0 and 1, and summing them together. The highlighted areas are enlarged: A) the Iquitos area, B) the northern Andes and Marañón area, and C) the Lima and Junín area, through to Cusco and Madre de Dios. The locations of selected cities and the capital, Lima, are shown in the large map, and their names given in maps A–C. IBAs which are included in the Peruvian protected area network are depicted with a vertical green dash, and those not included with a horizontal blue dash.

Figure 4 shows how the areas highlighted by different data coincide. All seven rasters were rescaled to values between 0 and 1, and summed together. The maximum value in the resulting raster was 4.64, meaning that the maximums of all seven rasters never wholly coincided. The highest values are found in the Chachapoyas – Utcubamba area (area 1), and together with the Marañón area (area 2) it also forms the largest continuous high-value area. The maximum numbers of both endemic bird species and bird species of conservation concern, as well as the distributions of bird species named by the respondents in the survey are found within this area. Area 1 was also visited by all tour companies included in our analysis. The highest values reached by each individual data within the highlighted areas are presented in Table 7.

**Table 7:** Highest values reached by each individual data within the areas highlighted in our study.

Area name	Bird spp. of conservation concern	Endemic bird spp.	Total number of bird spp.	Interviewees mentioning the area	Survey: distributions	Survey: localities	Tour companies
1. Chachapoyas - Utcubamba	13	23	368	2	5	2	5
2. Marañón	13	29	418	3	4	2	2
3. Olmos – Limón	5	6	136	1	4	2	5
4. Huánuco - Carpish	7	23	390	4	3	1	4
5. Junín	6	23	429	4	2	0	1
6. Satipo Road	3	16	355	2	1	1	4
7. Santa Eulalia - Marcapomacocha	3	15	140	5	3	3	4
8. Abra Málaga - Machu Picchu	6	17	366	4	2	2	5
9. Manu Road	8	14	407	4	2	2	5
10. Madre de Dios - Tambopata	2	1	623	6	3	2	5
11. Iquitos	2	2	602	6	2	0	3

The maximum values for each data are written in **bold**. The nation-wide maximum value for total species richness (666) was not reached within the highlighted areas.

The Olmos-Limón area (area 3) contains a high number of bird species mentioned by the birdwatchers, such as the White-winged Guan (*Penelope albipennis*) and Peruvian Plantcutter (*Phytotoma raimondii*) and was visited by all tour companies. It also holds a somewhat high number of bird species of conservation concern.

The Huánuco-Carpish area (area 4) has a high number of both endemic species and species of conservation concern, and was included in almost all of the tour companies' itineraries.

The Junín area (area 5) has also a high number of endemic species. The area highlighted by this data did not emerge as highly visited by the tour companies, but is very close to Lake Junín which was visited mainly for the Junín Grebe (*Podiceps taczanowskii*) and Junín Rail (*Laterallus tuerosi*).

Satipo Road (area 6) was visited by almost all tour companies and holds a relatively large number of endemic species.

The Santa-Eulalia – Marcapomacocha area (area 7) has a high number of species sought after by birdwatchers, and was visited by almost all tour companies. This area was also mentioned by nearly all interviewees, and holds a relatively large number of endemic species.

The Abra Málaga – Machu Picchu, Manu road and Madre de Dios – Tambopata areas (areas 8–10) were visited by all five tour companies, and were mentioned by most interviewees. They hold a large total number of species, but fewer endemics or species of conservation concern – except for the Machu Picchu – Abra Málaga area with slightly more endemics, and Manu road with slightly more species of conservation concern.

The Iquitos area (area 11) holds a high total number of bird species and was mentioned by nearly all interviewees, and it was included in most tour companies' itineraries.

The largest unprotected IBA within the areas highlighted in our data is in area 4, the IBA PE072: Carpish. Completely unprotected IBAs are found also within areas 3 (PE010: Bosques Secos de Salitral – Huarmaca – Olmos) and 7 (PE078: Marcapomacocha, PE079: Alto Valle Santa Eulalia-Milloc and PE080: Pampas Pucacocha y Curicocha). There are no IBAs exactly within areas 5 and 6, though area 5 is next to the protected IBA of Lake Junín, and three other protected areas (SN07: Pampa Hermosa, SH01: Chacamarca and BP03: Pui Pui) are found in the vicinity. Partially unprotected IBAs are found in all other highlighted areas.

## Discussion

We used the example of birds and tourism in Peru to demonstrate how conservation needs and tourism-based economic potential can be linked spatially. Our analysis was focused on a specific niche of tourism, birdwatching tourism. An assessment of the scope of this activity within Peru is problematic: customer numbers from birdwatching tour companies only reflect an unknown portion of this market, since it is likely that a significant part of tourists interested in birds travel independently, buying services such as accommodation, transport and guiding locally. On the other hand, according to airport surveys done in 2010, 19% of Peru's international tourists mentioned birdwatching as a motivation for their trip [22]. With a total of 2 299 200 international tourists arriving to the country in 2010 [23], this would amount to over 400 000 tourists interested in birds arriving in Peru in 2010. However, contrasting with an earlier estimate of a total of 1000 birdwatchers arriving in Peru in 2005 [24], it is obvious that these numbers must be taken with a grain of salt. They reflect in particular the wide variety among tourists who could be interested in birdwatching, ranging from casual bird observers to devoted twitchers. Keeping this in mind, birds form a significant part of a wider tourism product, also serving as an attraction among others for less specialized nature tourism, and are therefore an important asset for conservation-oriented tourism projects.

It is no news that in Peru most sites with greatest conservation need for birds are concentrated on the eastern slopes of the Andes [25], [26], nor that many of the country's IBA areas fall outside of the protected area network [14]. The identification of regions with high genetic or phylogenetic diversity and gaps in conservation area networks is however only one step in sustainable conservation. Peru's areas of high endemic and threatened bird species richness coincide with historically high pressures for land use and with other factors limiting successful conservation effort [27], [28]; indeed, also globally a large part of biodiversity hotspot areas are densely populated [29]. Traditionally the largest conservation areas have been established in remote wilderness regions; however, it is vital that conservation effort be directed also to areas near population centres [28]. Finding synergies between conservation goals and local level development initiatives is fundamental for both the short-term protection of important sites and their long-term conservation.

Our aim was to highlight the fact that a diversity of means now exists to carry out this kind of conservation, and furthermore, that many of these high-importance sites actually assemble the necessary features for local conservation efforts based on e.g. birdwatching tourism. In Peru a number of areas or sites combine particularly interesting sets of characteristics making them emerge as potential showcases for integration of local conservation and development goals.

According to our study, most unused potential in this sense can be found in Peru in the central and northern Andes. In this area there are several wholly or partially unprotected IBAs where bird species such as Marvellous Spatuletail (*Loddigesia mirabilis*), Andean Cock-of-the-rock (*Rupicola peruvianus*), White-winged Guan (*Penelope albipennis*) and Andean Condor (*Vultur gryphus*) are an important attraction for bird-based tourism. Southeastern rainforest areas in Cusco and Madre de Dios currently form the core of Peruvian nature and bird tourism but the highest potential for conservation through tourism is arguably found in the less well established circuits in the regions of Amazonas, La Libertad, Huánuco, and even relatively near Lima. It is however worth noticing that even in the core of Peru's most important tourism area, near the ruins of Machu Picchu (area 8 in Figure 4), there is an IBA which is largely unprotected, and the highest concentration of endemic bird species, as well as bird species of conservation concern, falls outside of protected areas (Figure 2, maps A and C). While our analysis focused on the presence of birds as attractions for tourism, the presence of additional attractions, natural or cultural, near or within the highlighted areas should also be taken into consideration. A demand for this can be seen also in the replies to the survey we conducted for birdwatchers, where 14 of the respondents identified the possibility to combine cultural sightseeing with birdwatching as one of Peru's strengths.

While we didn't include in our analysis data sets representing pure accessibility for reasons mentioned previously, we compared the areas highlighted in our study with a data set representing a measure of accessibility, travel time to major cities [21]. Most of the areas highlighted in our study are located in relatively well accessible regions, with five or less hours of travel time to major cities. The rainforest lodges especially in the lowland Amazonian areas of Madre de Dios are by nature more remote and require more time for travel. The comparison with the travel time data indicated possible issues with accessibility also within the Marañón area (area 2 in Figure 4).

Benefits from tourism is an often-mentioned form of ecosystem service provided by biodiversity [30]–[32], and related payments for ecosystem services [33] can be channeled through several mechanisms to enable local-level participation. The options for this type of innovations are constantly expanding in Peru and elsewhere [34]-[37]. In Peru there are several mechanisms that enable different kinds of actors such as rural communities, private individuals, NGO's or consortia between them to establish, through their own initiative, local conservation areas based on tourism activities. On public land, concessions can be leased for both conservation and ecotourism. On private or communal lands, conservation could be implemented through private conservation areas, or through a relatively new mechanism in Peru, conservation easement (*servidumbre ecológica*). It is a legal agreement between either landowners (e.g. private or communal) or a landowner and the state, where a landowner voluntarily restricts in some way land use in their territory in favor of another territory – the benefit could come in form of e.g. improved ecosystem services [36]. The first Peruvian conservation easement was created in 2005, but in other Latin American countries and elsewhere, there is a longer history for their use [38], [39].

Traditionally tourism in Peru has been very polarized, the most established destinations being found in the south [40]. This polarization is reflected in the distribution of tourism infrastructure, which can at least partially explain also the fact that in the survey we conducted for birdwatchers, the state of tourism infrastructure in Peru emerged as both a strength and a weakness. During recent years, however, the Peruvian Commission on the Promotion of Peru for export and tourism (PromPeru) has promoted birdwatching tourism and other types of tourism in the country through the Southern, Central and Northern tourism circuits. Local-level conservation can be seen to be on the rise in Peru, with a total of 34 private and 13 regional conservation areas established since 2001 [10]. The largest number of private conservation areas in Peru is currently found in the Cusco region (9), followed by Amazonas (6). The largest number of regional conservation areas is however found in Loreto region, and while the first Peruvian conservation easement was established in the Cusco region, the other two established since then are located in Amazonas.

The success of conservation efforts can be jeopardized by several factors, not least of which is the potential for armed conflict in areas where illicit cash crops, especially coca (*Erythroxylum coca* and *E. novogranatense*) are grown [27]. This is also related to the uneven distribution of tourism in Peru: for example the northern areas near the Marañón valley, as well as the Tingo María area in central Peru highlighted also in our study are regions of a strong history of coca-related and other social conflicts [27], and have only fairly recently opened up for tourism. In these areas the blocking of roads during strikes is common and reduces willingness to invest in tourism.

The mechanisms enabling conservation work are also all related to a number of important and politically sensitive issues such as land tenure, land use, and indigenous rights, which require careful consideration of local conditions. Depending on e.g. the local situation of land tenure, these types of projects could either be used by local communities to support land-ownership claims in the case of them being informal dwellers or to strengthen the position of legally established communities.

It should also be noted that when operating in ecologically sensitive areas, all actions should be based on sound planning. Work on the assessment of tourism's effect on birds, people, and the environment should be encouraged, and the creation of e.g. codes of conduct for tourists and tour operators should be an essential part of any tourism operation. It is especially important to pursue methods to continuously monitor any effects tourism might have on local population and the environment [41]. Possible negative effects of tourism projects include direct or indirect negative effects to nature in form of e.g. disturbance or littering. Tourism revenue itself or hopes to gain it might attract too many entrepreneurs along with too much touristic pressure to the area or encourage greenwashing [42]. However, the value of birdwatching tourism in this sense can be seen in the direct dependence of the preservation of the attraction on the conservation of high-quality habitat. Problems can also be caused by the unequal distribution of tourism revenue within the destinations or the leakage of tourism revenue away from the destination economy, which could cause tension between local actors and reduce their motivation to conserve the touristic attraction [43], [44] (but see [45]). These issues highlight the importance of active participation by local communities in the projects. All in all, the aforementioned initiatives and mechanisms should be studied and promoted as they have potential for both conservation and empowerment of local communities.

The approach we presented to integrate data on tourism opportunities and conservation need is applicable in several geographical and thematical contexts. Even though we gave the same weight to all data layers, optionally the data sets could be weighed to reflect their relative importance to the study question at hand.

## Supporting Information

Abstract S1 **Abstract in Spanish.**
(DOC)
