# Supplementary material for: Bird Diversity, Birdwatching Tourism and Conservation in Peru: A Geographic Analysis
Source: PLoS One. 2011 Nov 23;6(11):e26786. doi: 10.1371/journal.pone.0026786 (PMC3223161; doi:10.1371/journal.pone.0026786)
Supplement: Abstract S1 — Abstract in Spanish. (DOC) [file pone.0026786.s001.doc]

**Resumen: Diversidad de Aves, Turismo de Aves y Conservación en Perú: Un Análisis Geográfico**

En el contexto global de la pérdida de biodiversidad no sólo es importante evaluar las necesidades de conservación, por ejemplo a través del análisis de cobertura de áreas naturales protegidas, sino también es necesario buscar soluciones prácticas para la implementación de proyectos de conservación. El turismo social y ecológicamente sostenible puede ser una de estas alternativas. En este estudio presentamos una metodología para vincular espacialmente la necesidad de conservación y el potencial económico basado en turismo, utilizando el turismo de aves en Perú como ejemplo. En nuestro análisis, identificamos áreas en Perú con un alto potencial para este tipo de proyectos. Varias áreas en las regiones de los Andes del Norte y Centro, tanto como en la Amazonía baja de Madre de Dios y Loreto emergen como promisorias para este tipo de actividades. Las estrategias para implementar proyectos de conservación en estas zonas incluyen, por ejemplo, concesiones para la conservación y ecoturismo, áreas naturales protegidas privadas y servidumbres ecológicas. Algunos de estos mecanismos también ofrecen nuevas oportunidades a las comunidades locales para asegurar sus derechos de propiedad, acceso y usufructo de sus tierras tradicionales.
